# First Discovery of Beta-Sitosterol as a Novel Antiviral Agent against White Spot Syndrome Virus

**DOI:** 10.3390/ijms231810448

**Published:** 2022-09-09

**Authors:** Cheng Chen, Jing-Lei Shen, Chang-Shuai Liang, Zhong-Chen Sun, Hai-Feng Jiang

**Affiliations:** College of Animal Science and Technology, Northwest A&F University, Xinong Road 22nd, Xianyang 712100, China

**Keywords:** beta-sitosterol, white spot syndrome (WSS), *Pinellia ternata*, innate immune signaling pathways, anti-WSSV activity

## Abstract

The outbreak of white spot syndrome (WSS) is a looming challenge, due to dramatic losses to the crustacean aquaculture industry. However, at present, there are no prophylactic or therapeutic means to control this infectious viral disease. Here, we screened fifteen medicinal plants for their inhibitory activity on the white spot syndrome virus (WSSV), using red swamp crayfish (*Procambarus clarkii*) as a model species. The results showed that the crude extracts of *Pinellia ternata* (Thunb.) Breit. had the highest inhibitory effect (91.59%, 100 mg/kg) on WSSV proliferation, and its main component, beta-sitosterol, showed a much higher activity (95.79%, 50 mg/kg). Further, beta-sitosterol potently reduced (*p* < 0.01) viral loads and viral gene transcription levels in a concentration-dependent fashion, and significantly promoted the survival rate of WSSV-challenged crayfish (57.14%, 50 mg/kg). The co-incubation assay indicated that beta-sitosterol did not influence the infectivity of WSSV particles. Both pre- and post-treatment of beta-sitosterol exerted a significant inhibitory effect (*p* < 0.01) on the viral load in vivo. Mechanistically, beta-sitosterol not only interfered with the expression of viral genes (immediate early gene 1, *ie1*; DNA polymerase, *DNApol*) that are important in initiating WSSV transcription, but it also attenuated the hijacking of innate immune signaling pathways (Toll, IMD, and JAK/STAT pathways) by viral genes to block WSSV replication. Moreover, the expression of several antiviral immune, antioxidant, pro-inflammatory, and apoptosis-related genes changed significantly in beta-sitosterol-treated crayfish. Beta-sitosterol is a potent WSSV inhibitor and has the potential to be developed as an effective anti-WSSV agent against a WSS outbreak in crustacean aquaculture.

## 1. Introduction

The aquaculture industry has developed steadily for nearly half a century, making it one of the fastest-growing sectors of food production in the world [1]. A report by the Food and Agriculture Organization of the United Nations (FAO) showed that global aquaculture production increased from 73.8 million tons in 2014 to 82.1 million tons in 2018, accounting for 46 percent of total fish production [2]. Crustacean aquaculture is a crucial aquaculture sector and an important economic activity in many Asian countries, particularly in China. It also helps to diversify the income structure of countries with a large population [3]. However, due to the development of intensive aquaculture and the expansion of the scale of production, the crustacean aquaculture industry is under the threat of epidemic infectious diseases. Among these threats, white spot syndrome (WSS), which is caused by the white spot syndrome virus (WSSV), is of greatest concern, due to its enormous damage to the crustacean aquaculture industry [4,5].

WSSV is the only member of the Whispovirus genus in the *Nimaviridae* family. It is responsible for the destructive WSS in crustacean aquaculture (especially in shrimp aquaculture) over the past few decades [5,6]. The virus is particularly damaging to shrimp, with mortality rates approaching 100% after 3 to 10 days of infection [7]. Since its discovery in China in 1992 [8], WSSV has spread rapidly to all shrimp-farming regions in the world, including the Americas, Europe, and Africa [9,10,11]. Currently, WSSV is among the diseases that are notifiable to the World Organization for Animal Health (OIE) [12]. 

Unfortunately, the study of the pathogenesis of WSSV is currently limited, due to the lack of crustacean cell lines [6]. Cellular immune responses, such as phagocytosis and apoptosis, are generally considered to play an important role in host resistance to WSSV infection [3,10]. Several novel cellular events related to WSSV infection have been recently identified, including endocytosis and the intracellular transport of WSSV, innate immune pathways in response to WSSV infection, and the regulation of viral gene expression by host immune systems [3,6,10]. Despite these advances, many fundamental gaps in the pathogenesis of WSSV remain, along with a lack of effective treatments or control measures to combat WSSV outbreaks [13]. The complex transmission routes of infection and the wide host range of the virus make it difficult to completely remove the virus from affected water bodies [5]. Moreover, the immune protection conferred by crustacean vaccination remains controversial and vaccine development has come to an impasse [13]. Consequently, there is a pressing need to find prophylactic or therapeutic agents for the control of WSSV infection.

Various natural compounds that are present in herbal medicines have broad application prospects, due to their diverse pharmacological activities [14,15,16]. Herbal medicines have become an increasingly popular choice for viral disease prevention and treatment in aquaculture, due to their wide sources and low prices [9]. In recent years, more and more natural compounds from herbal medicines, with antiviral activity to various aquaculture species, have been reported. Therefore, it is feasible and necessary to screen, from herbal medicines, high-efficiency active monomers with antiviral activity. 

For example, arctigenin, isolated from *Arctium lappa*, has significant inhibitory activity against spring viraemia of the carp virus (SVCV) in common carp (*Cyprinus carpio*) [17], and *Prunella vulgaris* and its main constituent ursolic acid inhibit the proliferation of infectious hematopoietic necrosis virus (IHNV) in both *epithelioma papulosum cyprini* cells and rainbow trout (*Oncorhynchus mykiss*) [18]. Notably, our previous studies revealed that luteolin from *Lonicera japonica* [10] and genipin from *Gardenia jasminoides* Ellis have potent antiviral activity against WSSV in crayfish (*Procambarus clarkii*) [10,19]. Our antiviral screening of herbal medicines found that the crude extracts of *Pinellia ternata* (Thunb.) Breit. possess strong activity against WSSV. Beta-sitosterol (24-ethyl-5-cholestene-3-ol), a common phytosterol, has been reported as the main antiviral active ingredient of *P. ternate*, which exerts an inhibitory effect on herpes simplex virus Type 2 and human coronavirus NL63 and ameliorates influenza A virus-induced tissue damage in mice [20,21,22]. In addition, beta-sitosterol can regulate a variety of physiological functions (antioxidant, anti-inflammatory, antitumor, immunomodulatory, and antiasthmatic) and has broad application prospects [23]. However, the antiviral activity of beta-sitosterol against WSSV is yet to be explored. 

The present study was conducted to investigate the antiviral and protection effects of beta-sitosterol against WSSV, based on a crayfish (*P. clarkii*) model. Furthermore, we explored the antiviral mechanisms of beta-sitosterol in terms of infectivity, therapeutic and preventive effects, viral/host gene expression, and a biochemical parameter. Generally, the present study not only provides a new theoretical basis for the development of beta-sitosterol as an efficient and practicable anti-WSSV drug, but also provides a valuable reference for future research on the antiviral effects of beta-sitosterol on other aquatic animal viruses.

## 2. Results

### 2.1. In Vivo Antiviral Activity Screening of 15 Herbal Extracts

The result of antiviral activity screening is shown in Figure S2. Among the 15 herbal extracts, the inhibition rates of *Pinellia ternata* (Thunb.) Breit., *Amomum villosum* Lour. and *Chelidonium majus* Linn. exceeded 75%. The crude extracts of *Pinellia ternata* (Thunb.) Breit. showed the highest WSSV inhibitory activity (91.59 ± 3.18%, 100 mg/kg). Therefore, beta-sitosterol (CAS: 83-46-5) as the major active compound of *P. ternata* was chosen for further antiviral activity evaluation [24].

### 2.2. Beta-Sitosterol Inhibited the Replication of WSSV In Vivo

**Figure 1:**
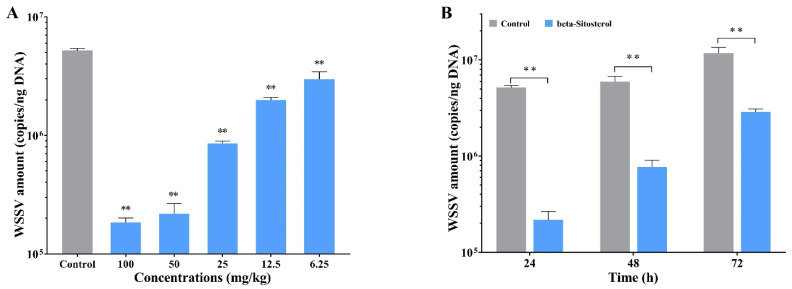
Beta-sitosterol inhibited the replication of WSSV in vivo. The experimental workflow is shown in Figure S3. (**A**) Beta-sitosterol with different concentrations (6.25, 12.5, 25, 50, and 100 mg/kg) had significant inhibitory effects on WSSV replication in crayfish (24 hpi). (**B**) Effects of beta-sitosterol (50 mg/kg) on WSSV replication at 24, 48, and 72 hpi. The data are shown as mean ± SD (n = 5). Asterisks indicate the significant difference between the experimental and control groups (** *p* < 0.01).

As illustrated in Figure 1A, beta-sitosterol with different concentrations had significant inhibitory effects on WSSV genomic DNA copy numbers in crayfish at 24 hpi. The results indicated that beta-sitosterol dramatically inhibited WSSV replication in a dose-dependent fashion. The highest and lowest inhibition rates for beta-sitosterol were at concentrations of 100 mg/kg and 6.25 mg/kg, with values of 42.45% and 96.44%, respectively. Furthermore, the inhibitory effects of beta-sitosterol on WSSV gradually decreased over time, but still showed a significant difference when compared with the control group (Figure 1B). The percentage inhibition of WSSV by beta-sitosterol (50 mg/kg) at 24, 48, and 72 hpi were 95.79%, 87.03%, and 75.44%, respectively. The results demonstrated that beta-sitosterol potently suppressed the replication of WSSV in vivo.

### 2.3. Beta-Sitosterol Inhibited the Transcription of WSSV Replication-Related Genes

**Figure 2:**
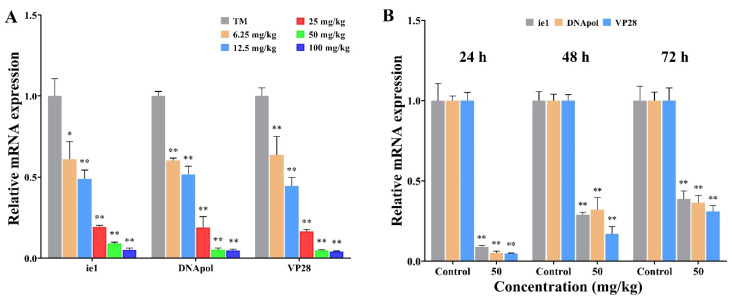
The effects of beta-sitosterol on the transcript levels of three vital viral genes (*ie1*, *DNA pol*, and *VP28*). The experimental workflow is described in Figure S3. (**A**) Influences of different beta-sitosterol contents (6.25, 12.5, 25, 50, and 100 mg/kg) on the transcript levels of WSSV genes at 24 hpi. (**B**) The transcript levels of WSSV genes were significantly inhibited by beta-sitosterol at 24, 48, and 72 hpi. The *18S* gene served as an internal reference. The data are shown as mean ± SD (n = 5). Asterisks indicate the significant difference between the experimental and control groups (* *p* < 0.05, ** *p* < 0.01).

The effects of beta-sitosterol on the expression of three vital viral genes (immediate early gene 1 (*ie1*), DNA polymerase (*DNApol*), and envelope protein 28 (*VP28*)) that are associated with WSSV replication are shown in Figure 2, and the experimental workflow is described in Figure S3. Compared with the control group, different concentrations of beta-sitosterol significantly inhibited the transcription levels of different genes (*ie1*, *DNA pol*, and *VP28*) of WSSV in crayfish at 24 hpi, and the inhibition effects tended to increase with the elevation of the beta-sitosterol concentration (Figure 2A). As shown in Figure 2B, the transcription levels of three important genes (*ie1*, *DNA pol*, and *VP28*) of WSSV were also significantly inhibited by beta-sitosterol at 24, 48, and 72 hpi (*p* < 0.01). These results indicated that beta-sitosterol acted on the suppression of the transcription of WSSV genes in vivo.

### 2.4. Beta-Sitosterol Provided Protection against WSSV-Infected Crayfish

**Figure 3:**
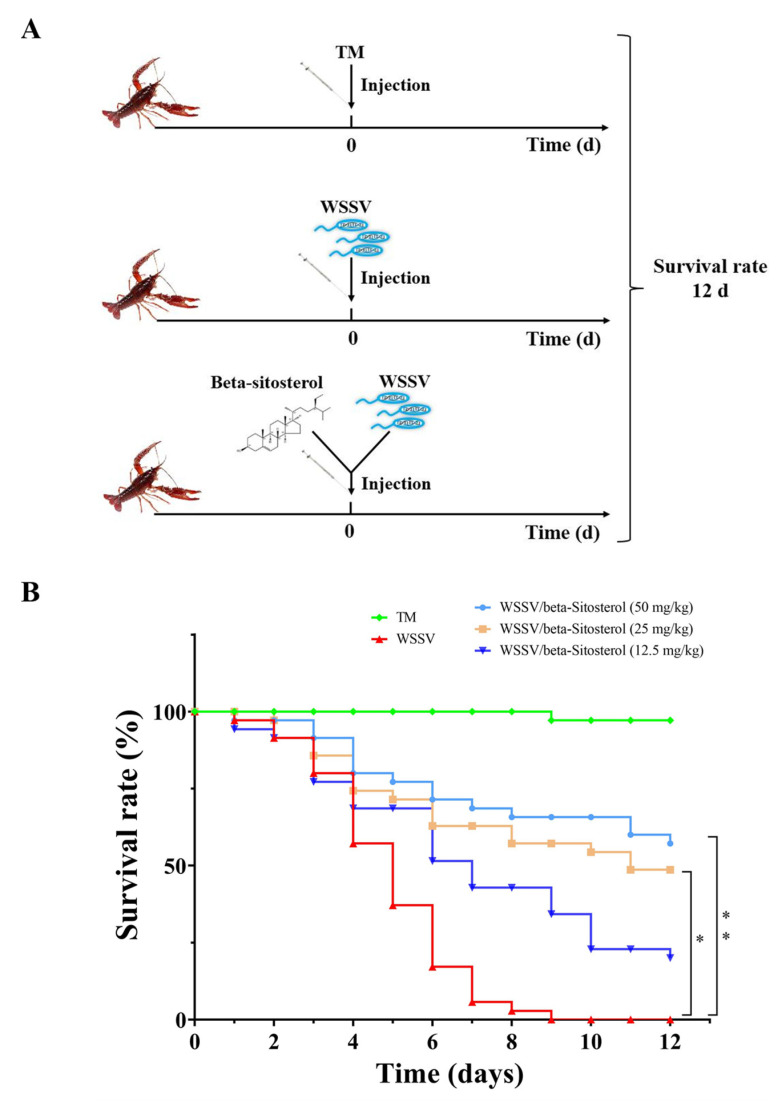
Beta-sitosterol increased the 12 day survival rate. (**A**) The experimental workflow. (**B**) The 12 day survivorship curves of crayfish in different groups. Asterisks indicate the significant difference between the experimental and control groups (* *p* < 0.05, ** *p* < 0.01).

The mixtures of WSSV suspension and various concentrations of beta-sitosterol (12.5, 25, and 50 mg/kg) were injected into crayfish to evaluate the protective effects of beta-sitosterol against WSSV infection. Figure 3 shows the survival curves of the different groups. The cumulative survival rate of the TM group (negative control group) for 10 days was 97.14%, while the cumulative mortality of the WSSV group (positive control group) reached 100% on the ninth day. In contrast, 25 mg/kg and 50 mg/kg of beta-sitosterol significantly reduced the mortality caused by WSSV, with survival rates of 48.57% and 57.14%, respectively (*p* < 0.05).

### 2.5. Beta-Sitosterol Displayed No Discernible Effect on Viral Infectivity

**Figure 4:**
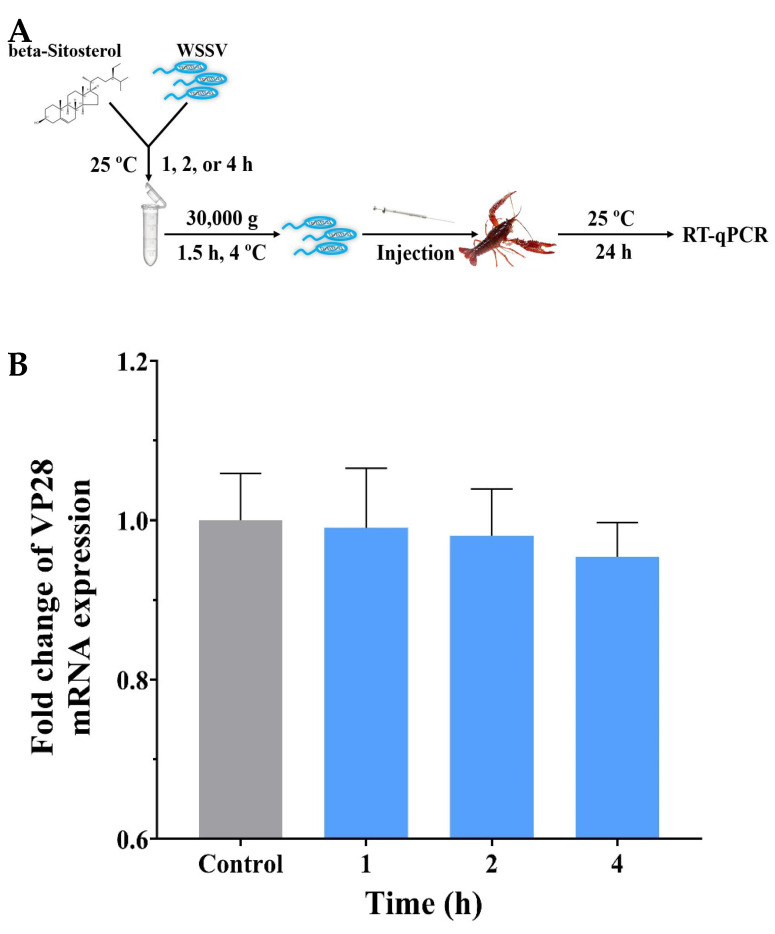
The effects of beta-sitosterol on WSSV infectivity in vitro. (**A**) The experimental workflow. (**B**) Detection of transcript levels of the *VP28* gene. The *18S* gene served as an internal reference. The data are shown as mean ± SD (n = 5).

An experiment was designed to investigate whether beta-sitosterol could directly affect WSSV infectivity (Figure 4A). The results are shown in Figure 4B. After 1, 2, and 4 h of co-incubation, the infection activity of WSSV decreased slightly but not significantly (*p* > 0.05). This experiment showed that beta-sitosterol did not directly affect the infectious activity of WSSV.

### 2.6. Beta-Sitosterol Conferred the Prophylactic and Therapeutic Effects against WSSV Infection In Vivo

**Figure 5:**
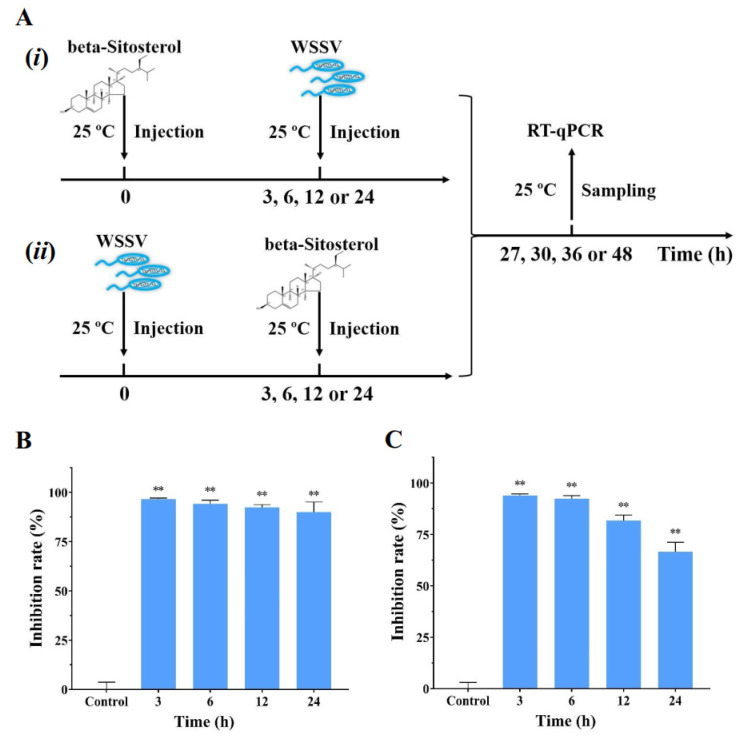
Beta-sitosterol conferred the prophylactic and therapeutic effects against WSSV infection in vivo. (**A**) (*i*) and (*ii*) are the workflows of (**B**,**C**), respectively. (**B**) The inhibition rate of beta-sitosterol (50 mg/kg) injected before WSSV infection at different times (3, 6, 12, and 24 h). (**C**) The inhibition rate of beta-sitosterol (50 mg/kg) injected after WSSV infection at different times (3, 6, 12, and 24 h). The WSSV-infected group was set as the control group. Data are shown as mean ± SD (n = 5). Asterisks indicate the significant difference between the experimental and control groups (** *p* < 0.01).

To evaluate the prophylactic and therapeutic efficacy of beta-sitosterol against WSSV infection in crayfish, we designed different experimental schemes (Figure 5A). As shown in Figure 5, it was clear that beta-sitosterol (50 mg/kg) possessed significant WSSV-suppressive activity, regardless of the treatment sequence. Figure 5B shows that the inhibition rates of beta-sitosterol prophylactic treatment for 3, 6, 12, and 24 h were basically the same. After WSSV infection, beta-sitosterol treatment showed a slight decrease in the inhibition rates at 12 and 24 h (Figure 5C), but still had significant inhibitory effects compared with the control group (*p* < 0.01). 

### 2.7. Beta-Sitosterol Reduced Total Protein Levels in hEMOLymph and Gill Tissues of Crayfish after WSSV Infection

**Figure 6:**
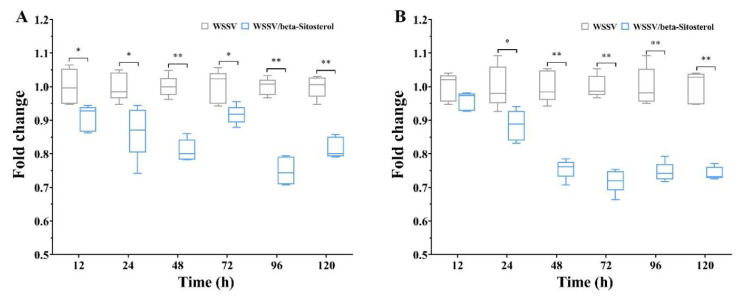
Beta-sitosterol (50 mg/kg) reduced total protein levels in hemolymph and gill tissues of crayfish after WSSV infection at different times (12, 24, 48, 72, 96, and 120 h). (**A**) Changes in total protein levels in gills under different treatments. (**B**) Changes in total protein levels in hemolymph under different treatments. The box plots show median value ± standard deviation and the interquartile range of likely variation (n = 5). Asterisks indicate the significant difference between the experimental and control groups (** *p* < 0.01, * *p* < 0.05).

The total protein levels are one of the indicators of the organism burden caused by viral infection. As shown in Figure 6A, the total protein levels in crayfish gills of the WSSV/beta-sitosterol group were significantly lower than those of the WSSV group at the detected time points (*p* < 0.05 for 12, 24, and 72 h, *p* < 0.01 for 48, 96, and 120 h). The total protein levels in hemolymph of the WSSV/beta-sitosterol group were lower than those of the WSSV group at the detected time points (*p* > 0.05 for 12 h, *p* < 0.05 for 24 h, *p* < 0.01 for 48, 72, 96, and 120 h) (Figure 6B). The results suggested that beta-sitosterol reduced the organism burden of WSSV-infected crayfish.

### 2.8. Beta-Sitosterol Treatment Modulated Innate Immune Signaling Pathways and Immune Factors

**Figure 7:**
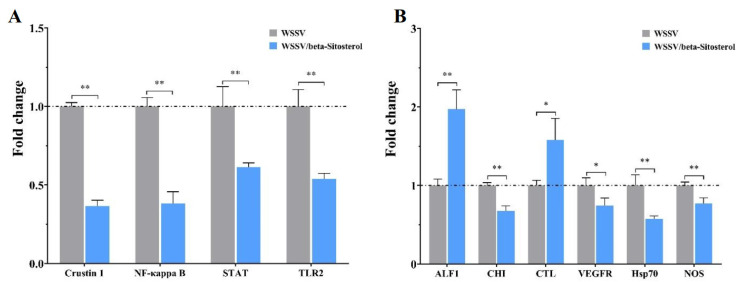
Bbeta-sitosterol regulated the transcription levels of innate immune signaling pathways and immune factor genes in crayfish infected with WSSV (50 mg/kg, 24 hpi). (**A**) Innate immune signaling pathways related genes (*Crustin 1*, *NF-κappa B*, *STAT*, and *TLR2*). (**B**) Other immune factor-related genes (ALF1, CHI, CTL, VEGFR, Hsp70, and NOS). The *18S* gene served as an internal reference. The data are shown as mean ± SD (n = 5). Asterisks indicate the significant difference between the experimental and control groups (* *p* < 0.05, ** *p* < 0.01).

To determine the effects of beta-sitosterol on the antiviral innate immune signaling pathways and immune factors, the transcription levels of related genes were determined in WSSV-infected crayfish (Figure 7). As illustrated by Figure 7A, compared with group crayfish, the transcript levels of *Crustin 1*, *NF-κappa B*, signal transducer and activator of transcription (*STAT*), and toll-like receptor 2 (*TLR2*) in the beta-sitosterol (50 mg/kg) treatment crayfish were significantly decreased 0.37-, 0.38-, 0.61-, and 0.54-fold, respectively, at 24 hpi (*p* < 0.01). The alterations of other immune-related factors are depicted in Figure 7B. Beta-sitosterol treatment significantly increased the transcription levels of the anti-lipopolysaccharide factor 1 (*ALF1*) and C-type agglutination (*CTL*) by 1.97- and 1.58-fold, when compared with the control group (*p* < 0.05). The transcription levels of chitinase (*CHI*), vascular endothelial growth factor receptor precursor (VEGFR), heat shock protein 70 (*Hsp70*), and nitric oxide synthase (*NOS*) were significantly decreased 0.67-, 0.74-, 0.57- and 0.77-fold, respectively, compared with the control group.

### 2.9. Beta-Sitosterol Regulated the Expression of Inflammatory-, Apoptosis-, and Antioxidant-Related Factors

**Figure 8:**
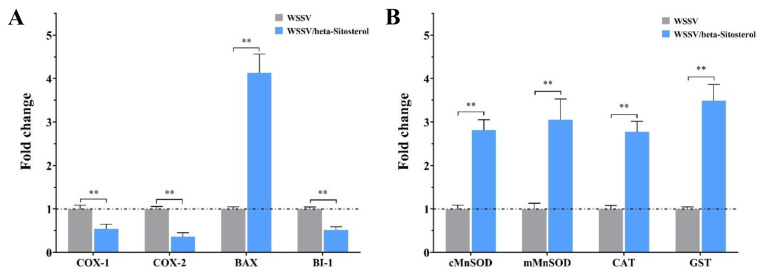
Beta-sitosterol regulated the expression of inflammatory-, apoptosis-, and antioxidant-related factors in vivo (50 mg/kg, 24 hpi). (**A**) Inflammation- and apoptosis-related genes (COX-1, COX-2, Bax and BI-1). (**B**) Antioxidant-related genes (cMnSOD, mMnSOD, CAT, and GST). The *18S* gene served as an internal reference. The data are shown as mean ± SD (n = 5). Asterisks indicate the significant difference between the experimental and control groups (** *p* < 0.01).

The effects of beta-sitosterol on the expression of inflammatory-, apoptosis-, and antioxidant-related factors in crayfish were detected by RT-qPCR. As shown in Figure 8A, in WSSV-infected crayfish, beta-sitosterol treatment caused a significant reduction in the expression of inflammatory-related factors cyclooxygenase-1 (COX-1) and cyclooxygenase-2 (COX-2) by 0.54- and 0.36-fold, respectively (*p* < 0.01). Beta-sitosterol treatment significantly increased the apoptosis-related factor BCL2-associated X (Bax) expression by 4.1-fold, while decreasing the expression of Bax inhibitor-1 by 0.52-fold (*p* < 0.01). As indicated in Figure 8B, the expression of antioxidant-related factors was significantly increased in beta-sitosterol treatment crayfish when compared with the control group (2.81-fold for superoxide dismutase cMnSOD, 3.05-fold for superoxide dismutase mMnSOD, 2.78-fold for catalase (CAT), and 3.49-fold for glutathione transferase (GST), *p* < 0.01).

## 3. Discussion

WSSV has caused great economic losses to the crustacean aquaculture industry, but there are no available licensed vaccines or antiviral drugs for the virus to date [4,5,13]. Many medicinal herbs and natural plant products have been found to demonstrate strong antiviral activity against various aquatic viruses, including WSSV [17,18,19,24,25,26], emphasizing the important role of herbal medicines as a starting point in the development of anti-WSSV drugs. In this study, we first found that *Pinellia ternata* (Thunb.) Breit. and its main active compound, beta-sitosterol, showed potent anti-WSSV activity. The inhibition effect of beta-sitosterol was comparable with that of genipin and luteolin (6.25 mg/kg to 50 mg/kg), which were reported to have good antiviral activity against crayfish WSSV infection in a previous study [10,19]. In addition, we verified the protective effect of beta-sitosterol treatment and explored the potential antiviral immunity mechanisms of beta-sitosterol.

Beta-sitosterol (24-ethyl-5-cholestene-3-ol) is a common phytosterol that is present in numerous Chinese medical plants. It has been shown to possess antiviral, antioxidant, anti-inflammatory, antitumor, immunomodulatory, and antiasthmatic functions [25,27]. However, studies on the antiviral activities of beta-sitosterol against aquatic viruses are scarce. The present study is the first to find that beta-sitosterol significantly suppressed the WSSV viral load and reduced the crayfish death that was invoked by WSSV infection. The experimental results showed that the co-incubation of beta-sitosterol with a WSSV solution did not influence the infectivity of WSSV. Moreover, beta-sitosterol showed a significant inhibitory effect, both before and after WSSV injection. These findings suggest that although beta-sitosterol did not inactivate WSSV in vitro or interfere the viral entrance, it did exert antiviral activity in vivo.

White spot syndrome virus (WSSV), an enveloped, circular, double-stranded DNA virus, possesses the largest genome (290 kb) of the animal viruses [28]. For large dsDNA viruses, the genes are usually differentially expressed in a cascading fashion manner, which means that the viral replication process relies on a continuous supply of replication factors from the previous stage [29]. According to the transcription pattern and the expression sequence of the genes, WSSV genes are generally categorized into three types: immediate early (IE) genes, early (E) genes, and late (L) genes [30]. Previous studies showed that IE genes, as a ubiquitous transcriptional regulator, had the function of simultaneously regulating the expression of E and L genes, and were considered to be involved in the initiation of the viral life cycle [29]. Therefore, it is now generally accepted that the IE and E genes are expressed before viral DNA replication, while the L genes expressed the structural proteins after virus replication to involve virus–host interactions [28,29]. Previous studies found that inhibiting the expression of WSSV IE genes (e.g., *ie1*) and E genes (e.g., *DNApol*) could reduce the expression of L genes (e.g., *VP28*) and further block viral DNA replication [29,31]. In this study, we found a dose-dependent inhibition on *ie1*, *DNApol*, and *VP28* genes expression in WSSV-infected crayfish (Figure 2), consistent with the reported inhibitory effect of beta-sitosterol on the expression of *DNApol* [32]. The downregulation of WSSV transcribes genes to the decreasing trend of viral genomic DNA copy numbers (Figure 1). Hence, we speculated that beta-sitosterol could inhibit the expression of regulators that are involved in initiating WSSV transcription (*ie1*, *DNApol*), thereby inhibiting the synthesis of viral structural proteins (*VP28*). This mechanism differs from that of luteolin, which blocks WSSV genome replication primarily by reducing the expression of viral gene transcription (*ie1*, *VP28*) [10].

Typically, pathogenic loads result in elevated protein levels in infected organisms. Polydnavirus was reported to increase protein concentrations in infected Manduca sexta larvae [33]. At the same time, a study demonstrated that WSSV could lead to increased protein and amino acid concentrations in the host [34]. Here, we found that, overall, the protein levels in the gill tissue and hemolymph of crayfish were lower than those in the virus group under the effect of beta-sitosterol. We observed that the protein levels in the gill tissue and hemolymph first decreased and then increased, relative to the WSSV group, which might be due to the weakening of the therapeutic effect of beta-sitosterol over time. These results indicated that beta-sitosterol helped to strengthen the immune system against WSSV infection, thereby reducing or normalizing protein levels in the host.

The toll pathway, the IMD pathway, and the JAK/STAT pathway are three main innate immune signaling pathways in crustaceans [35]. Among them, the Toll and IMD pathways regulate the expression of antimicrobial peptides (AMPs) against invading antigens via interaction with NF-κB [36]. As an important antimicrobial peptide, the RNAi of *Crustin 1* inhibited WSSV replication and reduced shrimp mortality, suggesting that WSSV might utilize *Crustin 1* to promote viral replication [37]. Toll-like receptors are one of the most well-known pattern recognition receptor family members that participate in the antiviral response against WSSV by inducing an anti-lipopolysaccharide factor in red claw crayfish *Cherax quadricarinatus* [38]. Studies found that NF-κB was activated by WSSV to promote WSSV replication in crustaceans [39,40]. Coincidentally, *STAT*, a key factor in the JAK/STAT pathway, was also hijacked by WSSV to activate the expression of the *ie1* gene to promote viral replication [41]. 

In addition to the antiviral immune pathways in crustaceans, several other important immune factors participate in the antiviral defense against WSSV. Chitinase is a chitin-degrading enzyme that is widely distributed in nature. It has been reported to promote WSSV infection in white leg shrimp *Litopenaeus vannamei* [42,43]. C-type-lectin and nitric oxide synthase showed antiviral and antibacterial activities in the *L. vannamei* [44,45]. In contrast, the vascular endothelial growth factor (VEGF) and heat shock protein 70 (Hsp70) could be hijacked by WSSV to facilitate a proliferation signaling pathway and played a key role in viral infection [46,47,48,49]. In this study, beta-sitosterol increased the expression levels of *ALF1* and *CTL* genes, while the expression levels of *Crustin 1*, *NF-κappa B*, *STAT*, *TLR2*, *CHI*, *Hsp70*, *VEGFR*, and *NOS* genes significantly decreased, suggesting that beta-sitosterol could, on the one hand, activate the host’s recognition of WSSV and induce the production of physiologically active substances to inhibit and eliminate pathogens, while on the other hand inhibiting the expression of some innate immune signaling pathways and immune factor genes that were hijacked by WSSV to promote virus replication, thereby inhibiting WSSV replication.

Virus-induced oxidative stress generates reactive oxygen species (ROS) and disrupts endogenous antioxidant defenses in host cells, leading to an imbalance in redox homeostasis [50]. Previous studies showed that WSSV infection reduced the activity of antioxidant enzymes and increased oxidative stress in *Exopalaemon carinicauda*, causing organ injury and systemic exhaustion, ultimately leading to the death of the shrimp [51,52]. Moreover, oxidative damage could also result in an inflammatory response by activating the pro-inflammatory cyclooxygenases [50]. Previous studies showed that beta-sitosterol displayed potent anti-inflammatory and antioxidant activities, which could block the activation of the NF-κb (nuclear factor kappa) signaling pathway to suppress the expression of inflammation-associated genes, thereby inhibiting the production of virus-induced pro-inflammatory factors [22,27]. In this work, we found a significant increased expression of three key antioxidant enzymes, SODs, CAT, and GST, whereas there was reduced expression of COX-1 and COX-2 in WSSV-beta-sitosterol treated crayfish (Figure 8). The results indicated that beta-sitosterol could balance the oxidative stress and inflammatory damage caused by WSSV infection.

Apoptosis is one of the important host defense mechanisms against virus infection [53]. Bax is a pro-apoptotic factor in the B-cell lymphoma-2 family that promotes host cell apoptosis by participating in the induction of the mitochondrial release of cytochromes [6]. Meanwhile, the expression of pro-apoptotic factors also enhances the anti-apoptotic response, and BI-1 protein is an anti-apoptotic factor that inhibits apoptosis [54,55]. It has been reported that reduced BI-1 gene expression by RNAi could inhibit WSSV replication in crayfish [54]. In this study, we found that beta-sitosterol treatment could promote the expression of the Bax gene and reduce the transcription level of the BI-1 gene in crayfish, indicating that beta-sitosterol promoted apoptosis. The results are consistent with the pro-apoptotic effect of beta-sitosterol [56]. Our findings suggest that beta-sitosterol could inhibit WSSV replication by inducing apoptosis during early antiviral immune responses.

## 4. Materials and Methods

### 4.1. Preliminary Screening of Antiviral Medicinal Plants

Fifteen medicinal plants purchased from local Chinese medicinal herb stores were screened, and their specific items of information, including plant names, medicinal parts, extraction solvents, safe concentrations and experimental concentrations, are shown in Table S1. The plant crude extracts were prepared according to our previous study [17,19]. Briefly, the purchased herbal medicine (50 g) was sufficiently pulverized and filtered through a 60-mesh sieve. Then, the crude extract of each herbal powder was soaked in 500 mL of methanol and extracted by ultrasonic reflux at 68 °C for 240 min, three times. Finally, after vacuum distillation and drying, the plant crude extracts were stored at 4 °C in the dark until use. The final extraction yield was approximately 6% to 16%.

### 4.2. Experimental Animals, Viral Inoculum, and Antiviral Compound

The experimental crayfish (*P. clarkii*, 13.51 ± 1.47 g) were obtained from the Xi’an seafood market (Shaanxi, China). The crayfish were temporarily kept in a sterilized and clean 70 L tanks with approximately 30 L water for seven days for acclimatization to the environment. During this period, the crayfish were fed once a day at noon with standard rearing conditions. Healthy crayfish were randomly selected for PCR detection with the primer *VP28*, as previously described [23]. The results showed that no positive was detected (Figure S1).

The WSSV isolates used in this study were the same as those that were used in our previous study [19,24], and were purified following the method described by Xie et al. Briefly, healthy crayfish were injected with a WSSV solution at the abdominal segment for virus multiplication. Approximately 15 g of muscle, gills, and other tissues of infected WSSV crayfish were ground, using tissue grinders (TissueLyser II, QIAGEN, Germany), and a 500 mL TNE buffer (100 mM Tris-HCl, 400 mM NaCl, 5 mM EDTA, pH 8.5) was added during grinding for dilution. The tissue homogenates were centrifuged at 3000× *g* for 15 min with supernatant filtered through a 400 mesh nylon sieve, then centrifuged at 9000× *g* for 15 min to obtain the supernatant. Following a centrifugation at 30,000× *g* for 5 min, the precipitation was discarded. Finally, after centrifugation at 30,000× *g* for 1.5 h, precipitation was obtained and resuspended in 10 mL of L-15 medium (Leibovitz’s L-15 with L-glutamine, Solarbio, Beijing, China) containing 6.5% fetal bovine serum (FBS) as the WSSV stock solution, which was stored at −80 °C until use. All centrifuge steps were done at 4 °C. Quantification of viral copy numbers followed previous studies [25]. The WSSV stock solution was diluted to 1.22 × 10^6^ copies/μL with the TM buffer. Beta-sitosterol (24-ethyl-5-cholestene-3-ol, CAS number 83-46-5, 95%) was purchased from Heowns Biochem Technologies, Llc. (Tianjin, China). The principles of laboratory animal care were followed throughout the duration of experiment and instructions were provided by the Animal Experimental Ethics Committee of Northwest A&F University.

### 4.3. Safety Assessment

The stock solution of the plant crude extracts and beta-sitosterol were prepared at 50 mg/mL in dimethyl sulfoxide (DMSO). After dilution by the TM buffer, healthy crayfish were randomly selected and injected in the abdomen with 100 μL of the working solutions of 200, 150, 100, and 80 mg/kg, respectively. The control group crayfish were injected with 100 uL TM buffer. There were three replications per treatment, with five crayfish per replicate (n = 15). The crayfish were observed for 48 h, and mortalities were recorded.

### 4.4. Antiviral Effect of Plant Extracts on WSSV In Vivo

In the anti-WSSV activity screening experiment of crude extracts, an equal mixture of the WSSV solution and the herbal extract solutions was used as the working solution and injected immediately after preparation at 25 °C. Crayfish were injected with 100 μL of the working solution (100 mg/kg or 50 mg/kg) and the control group crayfish were treated with 100 μL of the WSSV-TM mixture. Three replicates were performed for each group, with each group comprising five crayfish. The gill tissues from the crayfish were dissected after 24 h post-injection (hpi) and stored at −80 °C. The WSSV genomic copy numbers were determined, by quantitative real-time PCR (qRT-PCR), of the WSSV *VP28* gene according to our previous study [22], and then used to calculate the inhibition rate. The inhibition rate of WSSV infection was calculated with the formula: inhibition rate (%) = (control group − treatment group)/control group × 100. Beta-sitosterol, as the main bioactive compound of the *Pinellia ternate* (Thumb.) Breit., was selected for further investigation.

### 4.5. Antiviral Effect of Beta-Sitosterol on WSSV In Vivo

The antiviral effect of beta-sitosterol at different concentrations i.e., 0, 6.25, 12.5, 25, 50, and 100 mg/kg, were tested as per the protocol mentioned above. The effects of 50 mg/kg beta-sitosterol on WSSV replication at 24, 48, and 72 hpi were also determined. Three replicates were performed for each group, with each group comprising five crayfish. The gill tissues from the crayfish were dissected after 24 h post-injection (hpi) and stored at −80 °C. Crayfish and viral genes were determined by quantitative RT-PCR. A schematic representation of the workflow is provided in Figure S3.

### 4.6. Protective Effect of Beta-Sitosterol against WSSV Infection

To determine the protective effect of beta-sitosterol, crayfish of treatment groups were injected with 100 μL beta-sitosterol/WSSV solutions at different concentrations (12.5, 25, and 50 mg/kg). The control groups were treated with 100 μL vehicle solution (TM/DMSO) and TM/WSSV solution, respectively. There were 35 crayfish in each group. The survival of the crayfish was monitored daily for up to 12 days. 

### 4.7. The Effect of Beta-Sitosterol on Viral Infectivity In Vitro

The beta-sitosterol solutions (50 mg/mL) were incubated with WSSV solutions at 25 °C for 1, 2, and 4 h, respectively, and then the viral particles were separated from the mixture by ultracentrifugation (30,000× *g* for 1.5 h). Recovered viral particles were resuspended and injected into different groups of crayfish (100 μL). The crayfish in the control group were injected with WSSV/TM solution (100 μL), without centrifugation. Three replicates were performed for each group, with each group comprising five crayfish. The gill tissues from the crayfish were dissected after 24 h post-injection (hpi) and stored at −80 °C.

### 4.8. Antiviral Effect of Pre- or Post-Treatment of Beta-Sitosterol against WSSV

To test the preventive and therapeutic effect of beta-sitosterol, crayfish in different groups were injected with a beta-sitosterol solution (100 μL, 50 mg/kg) at 3, 6, 12, and 24 h before or after WSSV challenge, respectively. The crayfish in the control group were injected with the WSSV solution (100 μL) only at 0 h. The experimental grouping and sampling were the same as described above.

### 4.9. Total Protein Content Determination

Total protein was determined in the haemolymph and gill tissues of the WSSV-infected crayfish. The crayfish were injected with 100 μL mixed WSSV and beta-sitosterol at a concentration 50 mg/kg. The control groups were treated with a 100 μL WSSV-TM mixture. The haemolymph and gill tissues from the crayfish were sampled at 12, 24, 48, 72, 96, and 120 hpi, respectively. Hemolymph and anticoagulant (sodium citrate 0.05 mmol/mL, citric acid 0.025 mmol/mL, and glucose 0.08 mmol/mL) were mixed at a volume ratio of 1:1 to be tested. Gill tissues were ground into homogenate with an electric tissue grinder (G506001-001, ShengGong, Shanghai, China) at 4 °C and diluted to 1 mL with PBS. Samples were refrigerated at 4 °C and detected within 24 h. Total protein content was determined using BCA-protein assay (BCA Protein Assay Kit, Solarbio, Beijing, China). All measurements were performed on a multifunctional enzyme label instrument (SynergyH1, BioTek, Winooski, VT, USA).

### 4.10. Expression Analysis of Crayfish and Viral Genes

To further explore the mechanisms of beta-sitosterol, the expression of viral genes, i.e., immediate early gene 1 (*ie1*), DNA polymerase (*DNApol*), and envelope protein (*VP28*), were determined. The three genes are vital in the viral life cycle [57]. Meantime, the expressions of several crayfish genes that play important roles in innate immune signaling pathways were investigated, i.e., *Crustin 1*, *NF-κappa B*, signal transducer and activator of transcription (*STAT*), and toll-like receptor 2 (*TLR2*). Moreover, the expression of several important immune factors, i.e., anti-lipopolysaccharide factor 1 (*ALF1*), chitinase (*CHI*), C-type agglutination (*CTL*), vascular endothelial growth factor receptor precursor (*VEGFR*), heat shock protein 70 (*Hsp70*), and nitric oxide synthase (*NOS*), were determined. Furthermore, we examined the expression of several inflammatory-, apoptosis-, and antioxidative-related genes, i.e., cyclooxygenases *COX-1*, *COX-2*, BCL2-associated X (*Bax*), Bax inhibitor-1 (*BI-1*), catalase (*CAT*), glutathione transferase (*GST*), superoxide dismutase *cMnSOD*, and *mMnSOD*.

### 4.11. DNA/RNA Extraction, cDNA Amplification and qRT–PCR

The genomic DNA of the gill tissues was extracted using TIANamp DNA extraction kit (Tiangen, Beijing, China). The total RNA was extracted using RNAiso Plus reagent (Takara, Xi’an, China) and reverse transcribed into cDNA using a Hiscript^®^ Q RT Supermix for qPCR kit (+gDNA wiper) (Vazyme, Nanjing, China). The DNA and RNA concentrations were measured using a multifunctional enzyme label instrument (SynergyH1, BioTek, Winooski, VT, USA) and adjusted to 50 ng/μL. The qRT-PCR process was preformed using a ChamQ SYBR qPCR Master Mix kit (Vazyme, Nanjing, China) on LightCycler 96 (Roche, Switzerland). Reaction conditions were as follows: 95 °C × 5 min; followed by 42 cycles of 95 °C × 10 s and 55.5 °C × 30 s. All of the primers used in this study are listed in Table 1. The *18s*-rRNA gene was used as the reference gene, and the calculation of the target genes’ expression levels was conducted by the 2^−ΔΔCt^ relative quantification method [30].

### 4.12. Statistical Analysis

All data are presented as arithmetical mean ± standard deviation (SD) and a student’s *t*-test was used to determine the statistical significance of the difference. A log-rank (Mantel-Cox) test was used for the analysis of the Kaplan-Meier survival curves. The statistical analysis was conducted using IBM SPSS statistical software (version 20.0, Chicago, IL, USA). The value of *p* < 0.05 was considered statistically significant (* *p* < 0.05; ** *p* < 0.01).

## 5. Conclusions

In this study, beta-sitosterol was identified as the main active ingredient in *P. ternate* that inhibits WSSV replication. Beta-sitosterol effectively inhibits WSSV replication, exerts preventive and therapeutic effects, and provides a protective effect on WSSV-infected crayfish. Moreover, beta-sitosterol effectively inhibits WSSV replication, not only by suppressing important innate immune pathways that could be hijacked by WSSV, but also by regulating the expression of several key immune factors. In addition, beta-sitosterol could regulate a variety of antiviral immune factors, apoptosis factors, oxidative stress, and inflammatory damage caused by WSSV infection, and normalize protein levels, thereby inhibiting the replication of WSSV in crayfish. Beta-sitosterol is a promising agent for the control of WSSV infection in crustacean aquaculture. This research provides an important theoretical basis for the production of beta-sitosterol as a feasible and valuable anti-WSSV drug. Future work will further investigate the application of beta-sitosterol in large-scale production, with more intensive molecular biologic studies of the antiviral mechanism.

## Figures and Tables

**Table 1:** Primers used in this study.

Primer Name	Accession No.	Primer Sequences (from 5′ to 3′)	Size (bp)
*VP28*-F	KT995472.1	AAACCTCCGCATTCCTGTGA	141
*VP28*-R		TCCGCATCTTCTTCCTTCAT	
*ie1*-F	KT995472.1	GACTCTACAAATCTCTTTGCCA	283
*ie1*-R		TGCTGATAAACTCTTGAAGGAA	
*DNApol*-F	KT995472.1	CTCGCCAAAGTGAGTAGTGT	178
*DNApol*-R		CCTTGTTGATGGAGGTAGAA	
*Crustin 1*-F	GQ301201.1	CCACAGATGGCAATCGGAGTC	131
*Crustin 1*-R		AGGGAACGAACGCTGGAAAGT	
*NF-κappa B*-F	KF662471.1	TAGTGCGTGATGATGGGTCTT	136
*NF-κappa B*-R		GCTGATTATGGAGGCAGAAAA	
*STAT*-F		TGGTAGTGAAGAGAGGTTGAG	97
*STAT*-R		CATTGTTTCCCATCTGTCC	
*TLR2*-F	KP259728.1	AAGTCACTACGCAAACCA	102
*TLR2*-R		TACCACCATTTAGAGTAGACC	
*ALF1*-F	KU680792.1	CGGTTGGCGCCTCTACTACA	102
*ALF1*-R		GCGTGCTCGATGGCTCCTG	
*CHI*-F	FR990062.1	AATGGTGCTCAACCTCCT	151
*CHI*-R		CTGCGCTAAAGAAACAGAA	
*CTL*-F	KC857544.1	ACTTTGCTAACGCCAATCCAC	204
*CTL*-R		CTACGCTGTCATCGACGAACC	
*VEGFR*-F		AGTCGCCAGGAACCAGTG	
*VEGFR*-R		TGCCGAACCTAATGAAGATA	
*Hsp70*-F	DQ301506.1	GTTGACCAAGATGAAGGAGAC	100
*Hsp70*-R		CTGACGCTGAGAGTCGTTG	
*NOS*-F		TAATCCTTGACGGTGGTG	
*NOS*-R		TTGGCATCTTTCTTCTTCTC	
*COX-1*-F	KX268742.1	ATGGGATACCTCGACGTTATTC	202
*COX-1*-R		GCAGGAGGATAAGAATGCTGT	
*COX-2*-F	AF437613.1	GGTCATCAGTGATATTGAAGG	110
*COX-2*-R		TCTAATAAACGGAACCCAGAC	
*BAX*-F		TATAGTTGGCTCATTAGCAG	196
*BAX*-R		ATACTAAGTGAAGATGACTG	
*BI-1*-F		TGCCATTACATCTTGGGTTCT	157
*BI-1*-R		CGACCTAATCCCATCTCAAGC	
*cMnSOD*-F	EU254488.3	GCCACCACTAAAATACGAGTA	192
*cMnSOD*-R		CCATTGAACTTTATAGCTGGTA	
*mMnSOD*-F	KC333178.1	CATCACTCCAAGCACCACC	109
*mMnSOD*-R		GAGCAAGGGATATAACAGTAC	
*CAT*-F	KM068092.1	CGACCATACACCGCTTCAC	250
*CAT*-R		TTTCAGGAATGCGTTCTCTATC	
*GST*-F	HQ414581.1	ACTTAGAGACGGACTTCCAG	96
*GST*-R		CGAGGGCGAACTTCACGG	
*18S*-F	KX444578.1	ACCGATTGAATGATTTAGTGAG	153
*18S*-R		TACGGAAACCTTGTTACGAC	

## Data Availability

Data are contained within the article.

## Supplementary Materials

The supporting information can be downloaded at: https://www.mdpi.com/article/10.3390/ijms231810448/s1.
