# Supplementary material for: First Discovery of Beta-Sitosterol as a Novel Antiviral Agent against White Spot Syndrome Virus
Source: Int J Mol Sci. 2022 Sep 9;23(18):10448. doi: 10.3390/ijms231810448 (PMC9499679; doi:10.3390/ijms231810448)

Table S1. Specific information on medicinal plants used in this study, including plant names, medicinal parts, extracting solvents, safe concentrations, experimental concentrations.

Plants	Parts	Solvent	Safe con. (mg/kg)	Experimental con. (mg/kg)
Alpinia officinarum Hance	Rhizome	Methanol	>150	100
Amomum villosum Lour.	Fruit	Methanol	>150	100
Chelidonium majus Linn.	Herb	Methanol	>150	100
Fibraurea recisa Pierre	Stem and leaves	Methanol	>150	100
Iris dichotoma Pall.	Herb and roots	Methanol	>150	100
Leonurus japonicus Houttuyn	Herb	Methanol	>80	50
Linum usitatissimum Linn.	Seeds	Methanol	>200	100
Litsea mollis Hemsl.	Fruit and roots	Methanol	>150	100
Oxalis corniculata Linn.	Herb	Methanol	>200	100
Paederia foetida Linn.	Herb and roots	Methanol	>150	100
Pinellia ternata (Thunb.) Breit.	Tubers	Methanol	>150	100
Solanum nigrum Linne.	Herb	Methanol	>80	50
Humulus scandens (Lour.) Merr.	Herb	Methanol	>150	100
Urtica fissa E. Pritz.	Leaves	Methanol	>150	100
Xanthium strumarium Linne.	Fruit	Methanol	>80	50

Figure S1. Six crayfish were randomly selected for PCR detection (VP28, 141 bp), and the WSSV virus solution was used as a positive control. The results showed that these crayfish were in normal state.

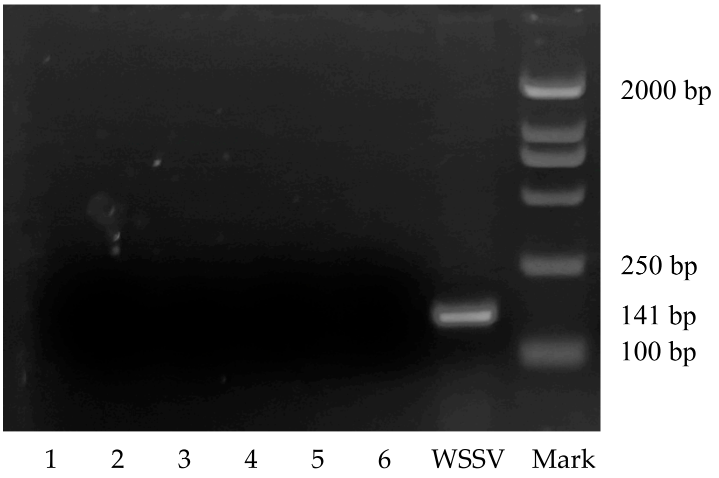

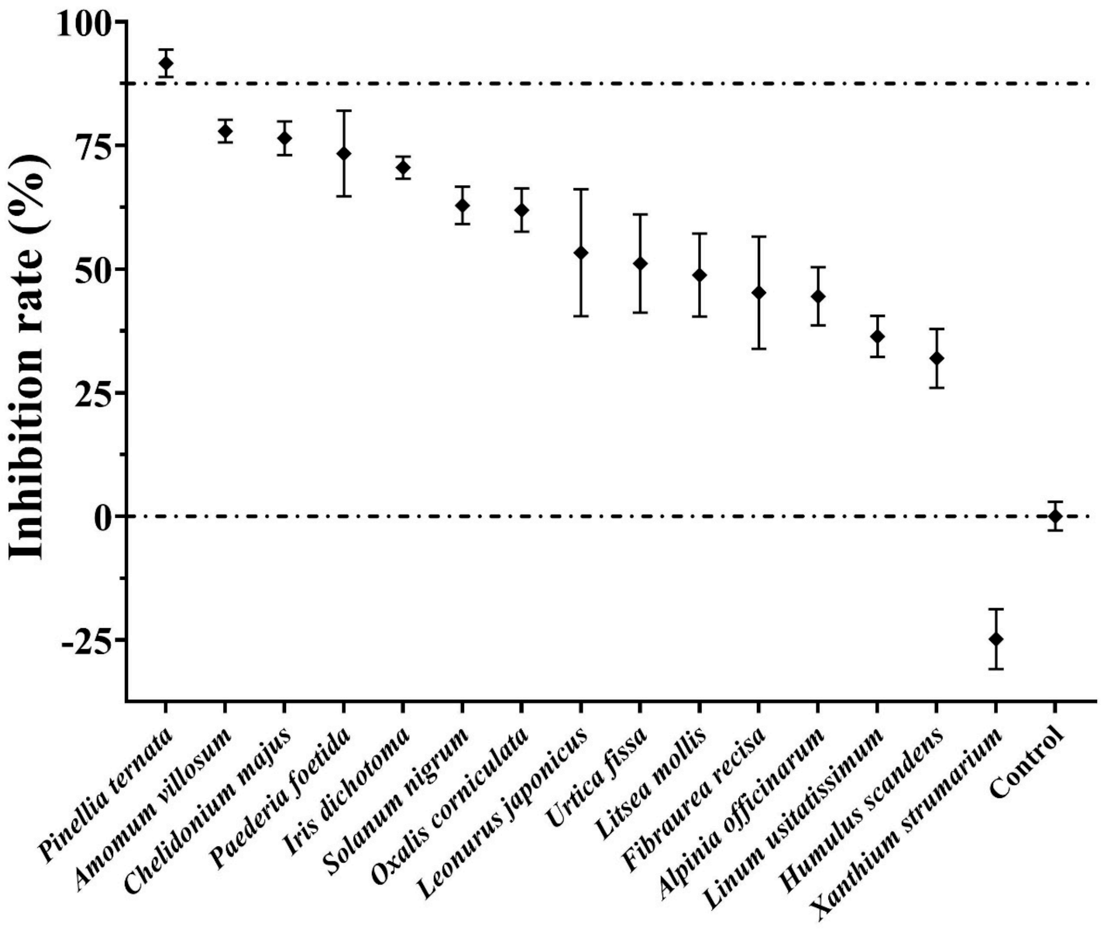

Figure S2. The inhibition rates of 15 different Chinese herbal extracts against WSSV were evaluated in vivo (100 mg/kg, 24hpi). The calculation formula was: inhibition rate = (control group - treatment group) /control group * 100 %. The data was shown as Mean ± SD (n=5).

Figure S3. The schematic depicting the experimental procedures in Figures 1 and 2.

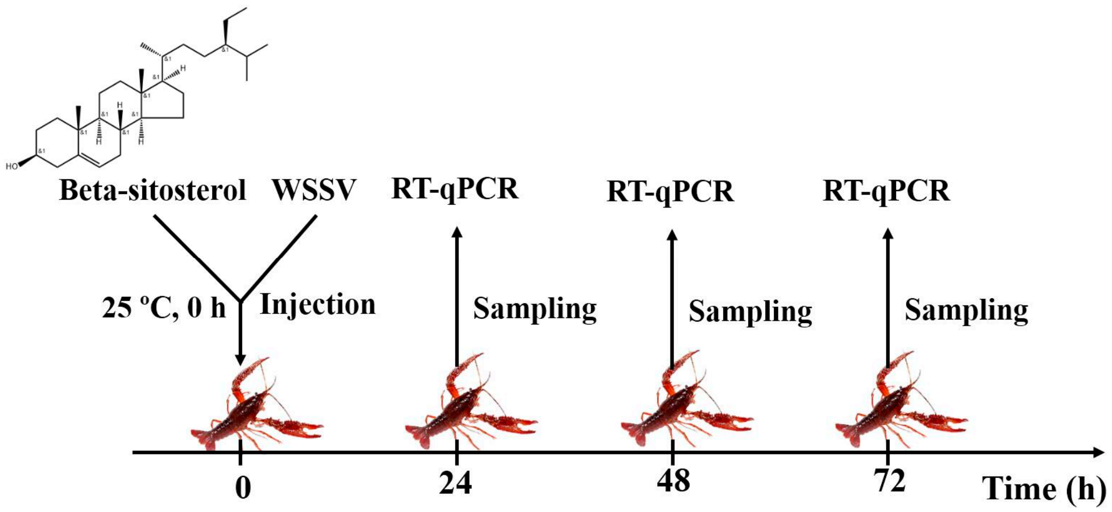

Supplement: Supplementary file 1 [file ijms-23-10448-s001.zip › ijms-1890856-supplementary.pdf]
